# A Newly Identified Passive Hyperaccumulator *Eucalyptus grandis* × *E*. *urophylla* under Manganese Stress

**DOI:** 10.1371/journal.pone.0136606

**Published:** 2015-09-01

**Authors:** Qingqing Xie, Zhenji Li, Limin Yang, Jing Lv, Timothy O. Jobe, Qiuquan Wang

**Affiliations:** 1 Department of Chemistry, the MOE Key Laboratory of Spectrochemical Analysis & Instrumentation, College of Chemistry and Chemical Engineering, Xiamen University, Xiamen 361005, China; 2 College of the Environment and Ecology, Xiamen University, Xiamen, 361102, China; 3 Boyce Thompson Institute for Plant Research, Ithaca, NY, 14853, United States of America; 4 State Key Laboratory of Marine Environmental Science, Xiamen University, Xiamen, 361005, China; Henan Agricultural Univerisity, CHINA

## Abstract

Manganese (Mn) is an essential micronutrient needed for plant growth and development, but can be toxic to plants in excess amounts. However, some plant species have detoxification mechanisms that allow them to accumulate Mn to levels that are normally toxic, a phenomenon known as hyperaccumulation. These species are excellent candidates for developing a cost-effective remediation strategy for Mn-polluted soils. In this study, we identified a new passive Mn-hyperaccumulator *Eucalyptus grandis* × *E*. *urophylla* during a field survey in southern China in July 2010. This hybrid can accumulate as much as 13,549 mg/kg DW Mn in its leaves. Our results from Scanning Electron Microscope (SEM) X-ray microanalysis indicate that Mn is distributed in the entire leaf and stem cross-section, especially in photosynthetic palisade, spongy mesophyll tissue, and stem xylem vessels. Results from size-exclusion chromatography coupled with ICP-MS (Inductively coupled plasma mass spectrometry) lead us to speculate that Mn associates with relatively high molecular weight proteins and low molecular weight organic acids, including tartaric acid, to avoid Mn toxicity. Our results provide experimental evidence that both proteins and organic acids play important roles in Mn detoxification in *Eucalyptus grandis* × *E*. *urophylla*. The key characteristics of *Eucalyptus grandis* × *E*. *urophylla* are an increased Mn translocation facilitated by transpiration through the xylem to the leaves and further distribution throughout the leaf tissues. Moreover, the Mn-speciation profile obtained for the first time in different cellular organelles of *Eucalyptus grandis* × *E*. *urophylla* suggested that different organelles have differential accumulating abilities and unique mechanisms for Mn-detoxification.

## Introduction

Manganese (Mn) is an essential nutrient for plant growth and development, known to act as a direct cofactor for about 35 plant enzymes so far [1]. For example, in chloroplasts, Mn is involved in the catalytic site of photosystem II (PS-II); it is also an important component of Mn superoxide dismutase, a key antioxidant enzyme in mitochondria [2, 3]. On the other hand, excess Mn may prevent plants from taking up and transporting other essential elements such as Ca, Mg, Fe and P, causing various negative effects in addition to toxicity[4]. Similarly, although Mn is a necessary nutritional element for mammals, including humans, participating in various metabolic and immune responses as well as reproduction [5, 6], it can cause neurodegenerative diseases [7, 8]. For example, elevated blood Mn levels were reported to be associated with decreased IQ scores in children and health deficits in newborns [9, 10]. Co-exposure with heavy metals such as lead, even at very low level, can further enhance Mn toxicity [11]. The main sources of Mn in the human population are diet, drinking water and inhalation [12], which are seriously affected by the environment, especially Mn-polluted soils [5]. Phytoremediation is considered a cost-effective and environmentally friendly technique for cleaning heavy metal polluted soils, and has attracted interest throughout the world [13]. Mn-hyperaccumulators possess tolerance mechanisms that allow them to detoxify and store high concentrations of Mn in their shoots. Chelation and compartmentalization are the most important detoxification mechanisms reported to date. Because tissue and subcellular localization of metals are dependent on plant species, general conclusions cannot be made based on the limited whole-leaf extractions and/or xylem sap analyses currently available. Furthermore, limited data exists regarding whether the sophisticated metal compartmentalization strategies used by some species changes under different environmental conditions. Although oxygen-containing donor ligands are considered crucial for Mn detoxification in some plants, the exact identity of these essential compounds has remained elusive until now.

Of the 492 presently known metal-hyperaccumulator species [14, 15], only 20 species are considered to be Mn-hyperaccumulators. These species are reported to have a foliar concentration of more than 10,000 mg/kg Mn by dry weight and are distributed in a highly restricted region of New Caledonia and Eastern Australia [14, 16–22]. Only 2 Mn-hyperaccumulators have been identified in China, *Phytolacca acinosa* Roxb [17] and *Schima superba* L[19]. Three additional species, *Polygonum hydropiper* L [23], *Polygonum perfoliatum* L [24] and *Polygonum pubescens* Blume [25], have been reported but need further confirmation. Recently, while screening field samples collected from a Mn mining area located in Liancheng, Southeastern China, we found that the hybrid *Eucalyptus grandis* × *E*. *urophylla* can hyperaccumulate Mn from Mn-polluted soils. It is a prominent species originally identified in Brazil [26] and imported to China in 1984. It is widely used in south China for timber and in the paper industry [27] due to its superior wood properties, rooting ability, rapid growth and disease resistance. In this study, we perform an in-depth investigation into the Mn accumulation and distribution in the hybrid *Eucalyptus grandis* × *E*. *urophylla*, and the possible detoxification and accumulation mechanisms.

## Materials and Methods

Soil and plant samples were collected from fields near abandoned mines open to public with no permission requirements.

### Plant and soil sample collection

The Liancheng mining area is located in Liancheng, Fujian Province, Southeastern China (25°44'00.00"N, 116°46'00.00"E), having a subtropical continental climate with an annual average temperature of 18.7°C, an average rainfall of 1191 mm/year and an average insolation of 1950 h/year. The Liancheng mining area is famous for its high production of Mn, Pb, and Zn. There are two main mine tailing sites, including the “Mn site” and the “Pb/Zn site” which were named by the abundance of Mn and Pb/Zn, respectively. During the field survey, we collected samples from the dominant plant species, including leaves, stems and host soils from both sites. Here we report data only for the Mn site. The Mn content of 23 plant species and their host soils was determined (Table 1). Mature leaf and stem samples of *Eucalyptus grandis* × *E*.*urophylla* from the mining area were collected from the lower canopy of 1–2 year-old plants. For each replicate, leaves from different stems were mixed together to obtain samples of similar weigh. Leaf and stem samples of *Eucalyptus grandis* × *E*.*urophylla* with the similar age were also collected from an uncontaminated area on the campus of Xiamen University, Xiamen, Fujian Province, Southeastern China (24°26'00.00"N, 118°04'00.00"E). This site has a subtropical continental climate with an average annual temperature of 20.6°C, average rainfall of 1315 mm/year and average insolation of 1953 h/year. All plant samples were washed thoroughly with Milli-Q water and frozen at –20°C prior to analysis. Soils were sieved with 100 mu mesh and air-dried before digestion.

**Table 1:** Manganese content in the soils, leaves and stems of the plant species from the Mn site at the Liangcheng mining area in southeastern China.

Plant Species	Family	Soil^a^	Leaf^b^	Stem^c^
*Eucalyptus grandis*×*E*.*urophylla* ^**CA**^	*Myrtaceae*	709 ± 136	640 ± 34	372 ± 34
*Eucalyptus grandis*×*E*.*urophylla*	*Myrtaceae*	13738 ± 2292	**13549± 1976**	**5231 ± 82**
*Neolitsea wushanica* (Chun) Merr	*Lauraceae*	12214 ± 1487	235 ± 24	127 ± 22
*Sapium sebiferum (L*.*)* Roxb	*Euphorbiaceae*	13578 ± 594	2354 ± 91	4716 ± 23
*Aphananthe aspera* (Thunb.) *Planch*	*Ulmacea*	14532 ± 799	1424 ± 101	256 ± 61
*Ligustrum sinense* Lour.	*Oleaceae*	15530 ± 187	4297 ± 202	1734 ± 254
*Smilax china* L.	*Liliaceae*	12615 ± 487	5673 ± 594	2603 ± 63
*Dennstaedtia wilfordii* (T. Moore) Christ	*Dennstaedtiaceae*	16796±2934	2042 ± 102	**14365 ± 659 (root)**
*Phytolacca acinosa* Roxb.	*Phytolaccaceae*	13782 ± 929	4142 ± 144	221 ± 21
*Miscanthus floridulus* (Labill.) Warburg ex K. Schumann	*Poaceae*	16896 ± 2034	1731 ± 92	3743 ± 436 (root)
*Pinus massoniana* Lamb.	*Pinaceae*	17789 ± 1523	**8305 ± 202**	3964 ± 86
*Polygonum chinense* L.	*Polygonaceae*	12415±2328	**10849 ± 5133**	2244 ± 314
*Pteris semipinnata* L.	*Pteridaceae*	13012±454	2687 ± 224	1774 ± 114
*Berchemia floribunda* (Wall.) Brongn.	*Rhamnaceae*	11449±860	5791 ± 642	1811 ± 73
*Jasminum lanceolarium* L.	*Oleaceae*	11639 ± 1024	1848± 324	1147 ± 178
*Polli japonica* L.	*Commelinaceae*	9156 ± 821	360 ± 22	811 ± 13
*Equisetum hiemale* L.	*Equisetaceae*	12479 ± 1072	143 ± 145	2752 ± 457 (root)
*Commelina bengalensis* L.	*Commelinaceae*	11654 ± 2312	878 ± 242	972 ± 75 (root)
*Eleutherococcus trifoliatus* L.	*Araliaceae*	10455 ± 1322	964 ± 13	—-^d^
*Paulownia kawakamii* L.	*Scrophulariaceae*	12533 ± 1527	1121 ± 449	676 ± 53
*Pteris fauriei* L.	*Pteridaceae*	12984 ± 556	864 ± 167	709 ± 103 (root)
*Woodwardia prolifera* L.	*Blechnacea*	9241 ± 378	833 ± 924	816 ± 93 (root)
*Houttuynia cordata* L.	*Saururaceae*	8819 ± 1002	3581 ± 564	3329 ± 777 (root)
*Argyreia seguinii* L.	*Convolvulaceae*	10998 ± 978	1721 ± 839	1295 ± 99

a, Mn concentration in the host soils of the dominant plant species (mg/kg DW), n = 3

b, Mn concentration in the leaves (mg/kg DW), n = 3

c, Mn concentration of the stem (mg/kg DW); d, no tissue was collected; CA: control area.

### Determination of metals with ICP-MS

Leaf and stem samples were dried at 105°C for 2 days, weighed, digested using a mixture of HNO_3_/HClO_4_ (5:1) and diluted with Milli-Q water prior to determining metal content using ICP-MS. Soil samples were pulverized, digested with a mixture of HNO_3_/HF/HClO_4_ (5:1:1) and analyzed by ICP-MS for Mn, Pb, As, Cu, Cd, Zn, and Co content [28]. Here we only report the Mn data. The operating conditions for the ICP/MS: ICP RF power was 1100 W; plasma gas flow was 16 L min^-1^; auxiliary gas flow 1.1 L min^-1^; nebulizer gas flow was 0.9 L min^-1^; isotope monitored was ^55^Mn; Dwell time was 100 ms.

### Plant culture and Mn treatment

Seeds obtained from NanHu Flower and Tree Market, Xiamen China, were surface sterilized with 70% EtOH for 1 min (twice), 100% EtOH for 1 min (twice) and washed with sterilized Milli-Q water (twice). The seeds were then germinated on one quarter-strength Murashige and Skoog (MS) agar plates for 4 weeks. Seedlings were transferred to half-strength Hoagland solution (2.5 mM Ca(NO_3_)_2_, 2.5 mM KNO_3_, 0.5 mM MgSO_4_, 0.5 mM KH_2_PO_4_, 40 μM Fe-EDTA, 25 μMH_3_BO_3_, 2.25 μM MnCl_2_, 1.9 μM ZnSO_4_, 0.15 μM CuSO_4_, and 0.05 μM (NH_4_)_6_Mo_7_O_24_, pH 5.8 to 6.0) for another 30 days. In addition to using 2.25 μM MnCl_2,_ we used 5, 5 × 10^2^, 5 × 10^3^, 1 × 10^4^ and 2 × 10^4^ μM MnCl_2_, respectively, for one-week Mn treatments [29]. After leaf, stem and root tissues were harvested, they were digested with HNO_3_/HClO_4_ (5:1) and diluted with Milli-Q water for Mn content measurement using ICP-MS.

### SEM X-ray microanalysis of metal distribution in stems and leaves

Sample preparation for SEM X-ray microanalysis was performed as previously described [30] with slightly modifications. Leaves and stems were mounted into small pieces approximately 5 mm × 5 mm and dehydrated using an ethanol series (30 min for each concentration: 10%, 30%, 50%, 70%, 95%, and 100%), 30 min in 100% acetone, and 30 min in iso-amylacetate. The samples were dried in a critical point dryer and coated with Au. Samples were then observed with a JSM-6390LV Scanning Electron Microscope. Elemental analyses were performed using X-ray microanalysis, and concentrations were expressed as percent by weigh.

### Subcellular fractionation method

The fractionation method for leaves was modified from the protocol described in [28, 31]. 0.5 g fresh leaves were homogenized using a mortar and pestle in an extraction buffer (0.25 M sucrose, 50 mM Tris-HCl, pH 7.5). Eight layers of gauze were used to strain the debris and express liquid from the residue. The residue mainly contained cell walls and was designated as the cell wall fraction. The liquid was then centrifuged at 600 g for 10 min. The pellet was collected and called the cell nucleus fraction. The resultant supernatant solution was then centrifuged at 2,000 g for 15 min, and the pellet was collected and called the chloroplast fraction. The supernatant was then centrifuged at 10,000 g for 20 min, the residue constituted the mitochondria fraction, while the supernatant retained was the ribosome containing fraction. All steps were performed at 4°C and all centrifuge steps were repeated twice. After suitable digestion and dilution, the Mn content of each fraction was determined using ICP-MS.

### Water soluble protein isolation

A soluble protein isolation method was used as described in [32, 33] with some modifications. Briefly, fresh plant tissue was weighed (20 g) and homogenized with a mortar and pestle in liquid nitrogen. We added 250 mL of extraction buffer containing 50 mM NH_4_HCO_3_, 1 mM phenylmethylsulfonyl fluoride (PMSF), 1 mM EDTA, 1 mM SDS, 20 mM β-mercaptoethanol to the powder. Next, four layers of gauze were used to filter the solution to remove residue. After centrifuging at 20,000 g at 4°C for 15 min, 100% ethanol was added to the supernatant to precipitate the proteins. To dissolve the pellets, a reasonable volume of 50 mM NH_4_HCO_3_ (pH 7.8) was added and centrifuged at 25,000 g at 4°C for 15 min. The supernatant was filtered using a cartridge with a 0.45-μM cellulose membrane and stored at - 20°C. A 100 μL aliquot of sample was used for HPLC/ICP-MS. The operating conditions were the same as described in [34]: (A) HPLC parameters: Superdex 75 10/300 GL (300 × 10 mm I.D., 13 μm) size exclusion column was used, whose optimum separation range was 3000–7,0000 Mr; column temperature was (25 ± 1) °C; mobile phase was 5 mmol L^-1^ Tris + 100 mmol L^-1^ NH_4_HCO_3_ (pH = 7.8); flow rate was 0.75 mL min^-1^; injection volume was 100 μL; detection was UV (214 and 280 nm) and ICP-MS; molecular standards were Glutathione (GSH, 307 Da, elution time was 28.3 min), β-lactoglobulin (BLG, 18300 Da, elution time was 16.3 min) and ovalbumin (OVA, 45000 Da, elution time was 13.8 min). (B) The operating conditions for the ICP/MS was the same as described above.

### Cell wall protein isolation

The cell wall fraction was isolated from leaves using the method described in [35] with minor modifications. Fresh plant tissue was weighed (20 g) and homogenized with a mortar and pestle in liquid nitrogen, and then immediately homogenized in 20 mL buffer (pH 7.5) containing 50 mM Tris-HCl, 50 mM NaCl, 0.05% Tween-20 and 1 mM phenylmethylsulphonyl fluoride (PMSF), and centrifuged at 5,000 g at 4°C for 20 min. The pellet was washed four times with 50 mM Tris-HCl (pH 7.5), and suspended in 1 M NaCl and incubated for 24 h at 4°C. After which, it was centrifuged at 15,000 g under 4°C for 20 min. The supernatant was referred to as ionically linked cell wall proteins (ionically bound to the glycan network). The resulting pellet was washed with 1 M NaCl and rinsed 4 times with Milli-Q water. To release the covalently linked proteins (covalently bound to the glycan network), the pellet was washed further with 0.5% cellulase and 2.5% pectinase at 4°C for 24 h, and centrifuged at 15,000 g and 4°C for 20 min. The supernatant was referred to as covalently bound proteins. After filtration with a 0.45-μM cellulose membrane, 100 μL of sample was injected for HPLC/ICP-MS analysis. The operating conditions for the HPLC/ICP-MS were the same as described above.

### Chloroplast protein isolation

The chloroplast isolation method described in [28] was used with modifications. Briefly, 20 g of fresh leaves were pulverized to a fine powder in liquid nitrogen and homogenized in 50 mL chloroplast extraction buffer (0.33 M sorbitol, 50 mM methyl ester sulfonate sodium (MES), 2 mM MgCl_2_, 10 mM NaCl, 2 mM EDTA, 0.5 mM KH_2_PO_4_ and 2 mM sodium ascorbate). The slurry was filtered through four layers of gauze. The suspension was centrifuged at 1,000 g at 4°C for 2 min. The pellet (chloroplast) was suspended and osmotically broken in 5 mM MgCl_2_ for 30 seconds. Suspension buffer was added (0.66 M sorbitol, 40 mM MES, and 5 mM MgCl_2_) to the broken chloroplast fraction and centrifuged at 3,000 g at 4°C for 5 min. Chloroplast membrane was in the supernatant. The pellet (thylakoid) was resuspended in buffer containing 2 mM MES, 5 mM MgCl_2_ and 15 mM NaCl) and stored on ice for 1.5 h. We then took a small amount of thylakoid, and used 80% acetone to extract chlorophyll and measure chlorophyll as described in [36]. Next, we slowly added 20% Triton X-100 to the rest of the thylakoids (Triton X-100 (v): chlorophyll (g) = 25: 1) while slowly string on ice in the dark. We centrifuged the thylakoid at 4,000 g at 4°C for 30 min. The pellet was PS II, and the supernatant was PS I. The PS I and PS II fractions were stored in -20°C and 100 μL of sample was injected for HPLC/ICP-MS analysis. The operating conditions for the HPLC/ICP-MS were the same as described above.

### Vacuole protein isolation

We use the vacuole protein isolation protocol described in [37] with the modifications. Leaves (20 g) were surface sterilized with 70% EtOH. The lower epidermal layers were peeled off and the leaves were incubated in protoplast isolation buffer (0.6 M mannitol, 2% (w/v) cellulase, 0.5% (w/v) pectinase, 25 mM MES, pH 5.5) at 28°C. After 6 h, protoplasts were filtered through four layers of gauze and the suspension was centrifuged at 1,200 g for 1 min. The protoplast pellet was washed twice with a rinsing buffer (0.7 M mannitol, 10 mM Tris-HCl, 15 mM MES, pH 7.5) and resuspended in the rinsing buffer. The suspended liquid was applied onto a 21% (w/v) sucrose solution, centrifuged at 1,000 g for 10 min and the upper layer of supernatant (containing the protoplasts) was carefully removed and placed into a new tube.

Lysis buffer I (0.2 M mannitol, 10% (w/v) Ficoll 400, 20 mM EDTA, 5 mM HEPES-KOH, pH 7.5) at 42°C was prepared. The protoplast suspension prepared above was diluted 4-fold in lysis buffer I for 15 min. The suspension was loaded at the bottom of a centrifuge tube and covered with a mixture (1:2, buffer I: buffer II) (buffer II: 0.4 M betaine, 30 mM KCl, 20 mM HEPESKOH, pH 7.5), which is two times the volume of the above suspension, then covered with buffer II, which is one time the volume of the suspension. We centrifuged the gradient at 1,500 g for 20 min, and vacuoles were collected at the interface of the first and second layers. The vacuoles were concentrated by centrifugation at 1,800 g for 20 min, and then used to extract soluble protein as described above for HPLC/ICP-MS analysis. The operating conditions for the HPLC/ICP-MS were the same as described above.

### Preparation of tissue extracts for organic acid analysis

Organic acids were extracted using the method described in [38]. A 100mg pulverized leaf sample was prepared with liquid nitrogen and 5 mL 25 mM HCl were added in a 25 mL beaker, sonicated for 10 min, and 1 min vortex. The extraction was performed at ambient conditions and the procedure was repeated three times. The extracts from each subsample were pooled and centrifuged at 13,000 g for 15 min. The final volume of the supernatant was diluted to 5 mL with 25 mM HCl. Organic acids (including oxalic, tartaric, formic, malic, lactic, acetic, maleic, citric, fumaric and succinic acids) were analyzed by HPLC using the modified method described by [39] and the HPLC parameters for organic acid measurement were as the following: Aglient ZORBAX SB-C18 (4.6 mm I.D. × 250 mm in length; 5 μm) was used; column temperature was (25 ± 1) °C; mobile phase was 97% 25 mM H_3_PO_4_ and 3% methanol, (pH 2.5); flow rate was 0.7 mL min^-1^; injection volume was 100 μL and detection was UV (210 nm).

## Results and Discussion

### Mn concentration in soil, leaf and stem samples

**Fig 1:**
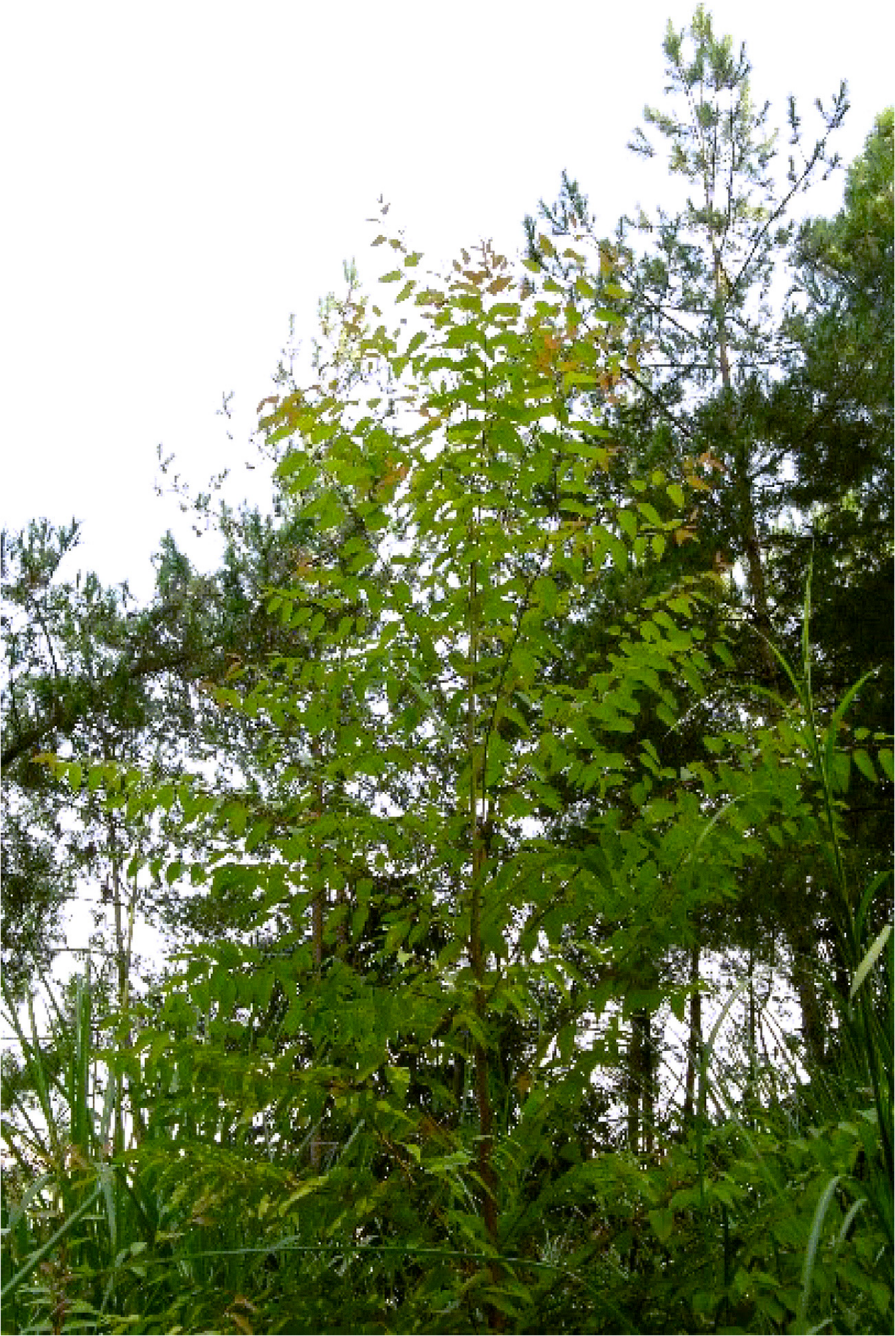
*Eucalyptus grandis* × *E*. *urophylla* growing at Liancheng manganese tailings in Southern China.

During a field survey of plant species in the mine tailing area in July, 2010, twenty-three dominant species were identified. Samples were collected and labeled using the family name, which are listed in (Table 1). We determined the Mn concentration in the host soil, leaves and stems as well as some roots of the plants. The Mn content in the soils ranged from 8,819 to 17,788 mg/kg at the Mn site, which is much higher than in normal soils (450–4,000 mg/kg) [40, 41]. The Mn concentration in plant tissues varied by species. With the exception of *Neolitsea wushanica* and *Polli japonica*, which contained less than 500 mg/kg Mn, most of the dominant plants accumulated Mn in the range of 1,000 to 5,000 mg/kg, which is much higher than those growing in normal soil, which accumulated 20–500 Mn μg/g dry weight [40–42]. Interestingly, among the 23 dominant species identified, the following four plant species showed extremely high Mn accumulation in their tissues: 8,305 mg/kg in the leaves of *Pinus massoniana* Lamb, 10,849 mg/kg in the leaves of *Polygonum chinense* L, 13,549 mg/kg in the leaves of *Eucalyptus grandis* × *E*.*urophylla*, and 14,365 mg/kg in the roots of *Dennstaedtia wilfordii*. Of these four plants, the hybrid *Eucalyptus grandis* × *E*. *urophylla* is a woody plant species having higher biomass. A 1–2 year old *Eucalyptus grandis* × *E*. *urophylla* growing at the Mn site is shown in (Fig 1). A summary of the leaf morphological characteristics, including 13–20 cm petioles, is shown in (Fig 1). Notice that the leaf blade is broadly lanceolate or ovate, thinly leathery, adaxially dark green and slightly glossy, silver white in the back with evident mid-vein and inconspicuous lateral veins. This species was growing well at the Mn site, accumulating 13,549 mg/kg DW in the leaves and 5231 mg/kg DW in the stems showing no signs of Mn toxicity. Thus, *Eucalyptus grandis* × *E*. *urophylla* should be considered a candidate for phytoremediation of Mn-polluted soils.

Interestingly, the Mn content in the leaves and stems of similarly aged *Eucalyptus grandis* × *E*. *urophylla* collected from the control area on the campus of Xiamen University (the host soil Mn content is 709 ± 136 mg/kg DW), was only 640 ± 34 mg/kg DW and 372 ± 34 mg/kg (Table 1). This suggests that *Eucalyptus grandis* × *E*. *urophylla* does not actively accumulate Mn when grown on normal soils unlike other Mn hyperaccumulators like *Triticum aestivum* L., which can accumulate over 1,000 μg/g [40, 41]. In this regard, *Eucalyptus grandis* × *E*. *urophylla* is a inducible Mn-hyperaccumulator under Mn-stress. Thus, we focused on this Mn-hyperaccumulator to investigate the physiological changes that occur at high Mn exposure levels and to understand the possible Mn accumulation and detoxification mechanisms before using this Mn-hyperaccumulator for the phytoremediation of Mn-polluted soils.

### Mn toxicity in *Eucalyptus grandis* × *E*. *urophylla*

**Fig 2:**
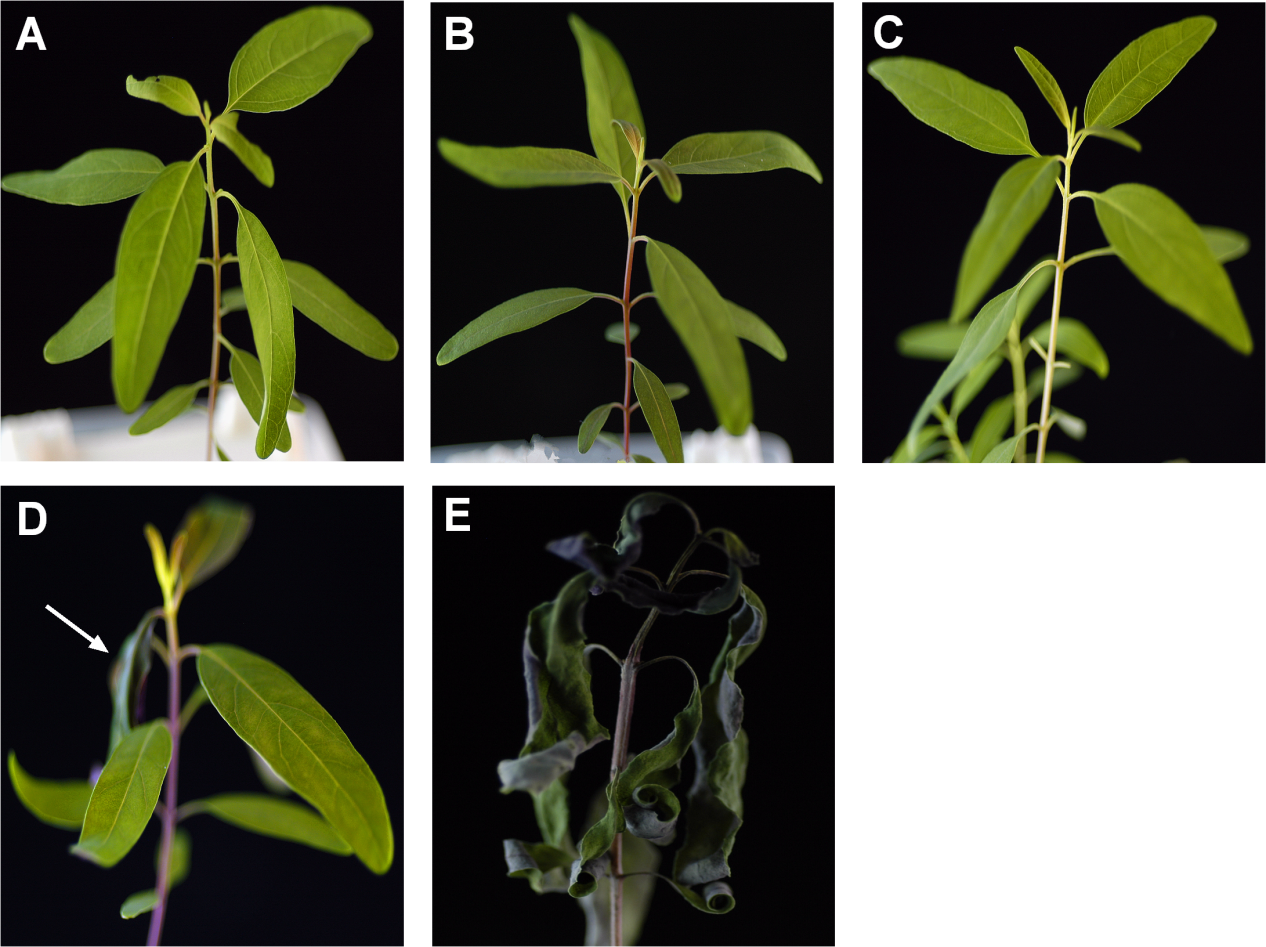
Observed Mn toxicity in *Eucalyptus grandis* × *E*. *urophylla*. Seedlings were treated in hydroponic solution with different levels of Mn for one week. A, the control Hoagland solution plus (5 μM); B, 500 μM; C, 5×10^3^ μM; D, 10×10^3^ μM; E, 20×10^3^ μM Mn. The white arrow in D indicated young leaves which turned purple, curled and crinkling symptoms under 10×10^3^ μM Mn treatment.

Seeds of *Eucalyptus grandis* × *E*.*urophylla* were surface sterilized and grown in a hydroponic system. Two-month-old seedlings were treated with different Mn concentrations for one week. The seedlings grew normally in Hogaland solutions containing 5 to 5,000 μM Mn without showing toxicity symptoms (Fig 2A, 2B and 2C); however, when exposed to 10,000 μM Mn, toxicity was observed in the seedlings. More specifically, only one or two young leaves turned purple, curled and crinkling symptoms were observed but the other leaves seemed normal and the whole seedling did not show impaired growth compared to lower Mn treatments (Fig 2D). When exposed to 20, 000 μM Mn (corresponding to 1,098.8 mg/kg), all the leaves showed strong symptoms of Mn toxicity with most of them turning purple and curling (Fig 2E). The critical concentration for Mn toxicity varies between plant species, but most plants show toxicity symptoms at Mn concentrations of 160–200 mg/kg [43]. Our results suggest that *Eucalyptus grandis* × *E*. *urophylla* can tolerate up to 10, 000 μM Mn (corresponding to 549.4 mg/kg) making its Mn tolerance ability similar to another Mn hyper-accumulator previously found in China, *Phytolacca acinosa* Roxb [29]. In a previous report, the acid-extractable Mn in soil from the Liancheng mining area accounts for 0.46 to 1.3% (wt/wt) [44]. Based on the total Mn concentration in the soils collected near *Eucalyptus grandis* × *E*. *urophylla* plants, the acid-extractable Mn ranged from 63.2 to 178.6 mg/kg. We are aware that the acid-extractable Mn in soil is much higher than the bioavailable Mn. However, if the threshold Mn concentration is 10,000 μM (corresponding to 549.4 mg/kg) based on the results of the hydroponic exposure experiments, the maximum Mn tolerance capacity of *Eucalyptus grandis* × *E*. *urophylla* has not yet been reached in the soil. Based on these findings, *Eucalyptus grandis* × *E*. *urophylla* should be a good candidate for remediating soils with extremely toxic amounts of Mn and may also be a good candidate for phyto-mining of Mn.

### Mn accumulation and distribution in *Eucalyptus grandis × E*. *urophylla*

**Fig 3:**
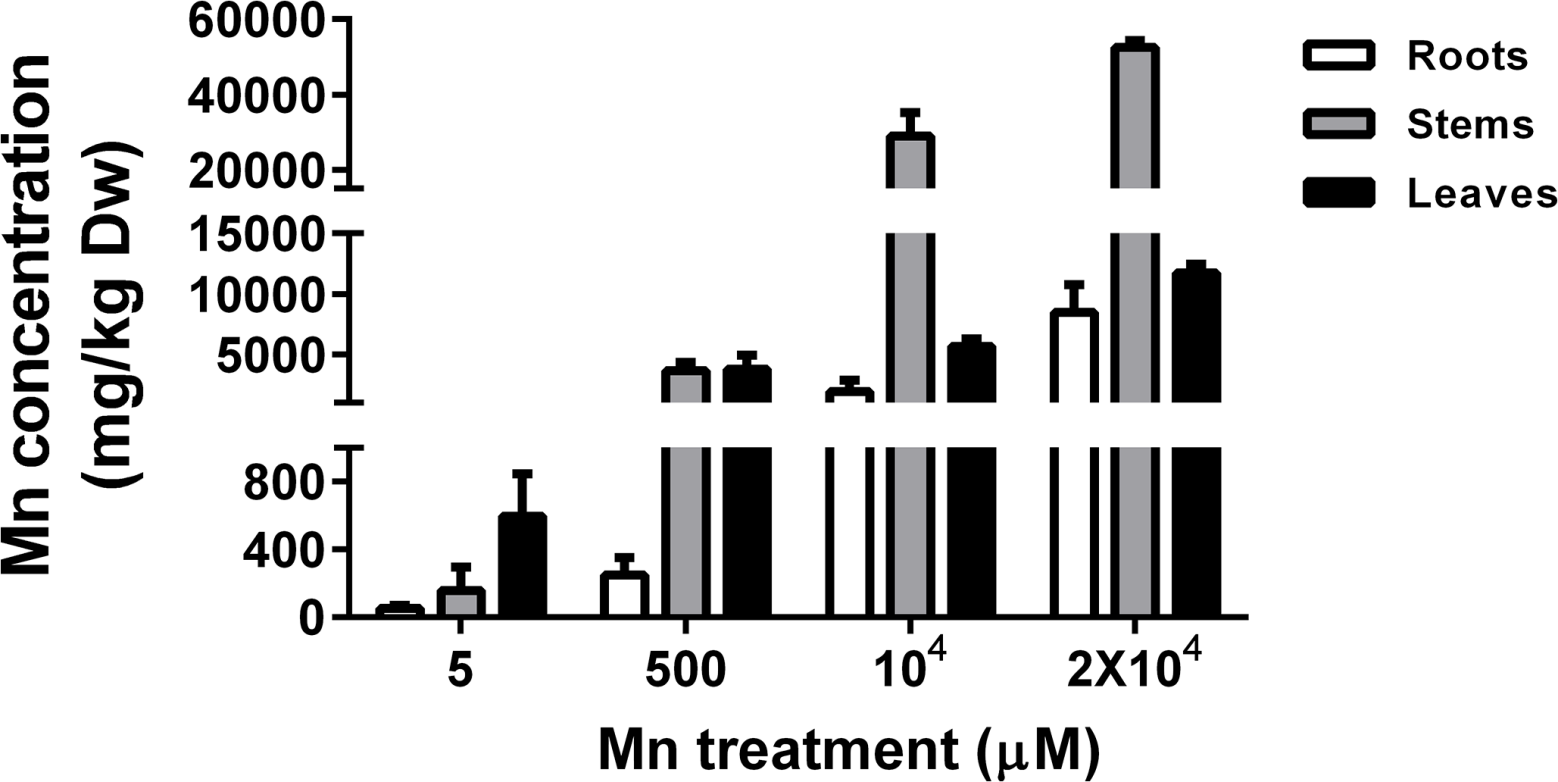
Accumulation of Mn in the roots, stems and leaves of *Eucalyptus grandis* × *E*. *urophylla*. Seedlings were treated with different Mn treatments (5, 500, 10^4^ and 2×10^4^ μM) in hydroponic solution for one week. Mn content in different tissues was measured with ICP-OES. Data represents mean ± SE. (n = 3).

We measured Mn concentrations in the roots, stems and leaves of Mn-stressed *Eucalyptus grandis* × *E*. *urophylla* using ICP-MS. The Mn distribution in different tissues is shown in (Fig 3). The plant accumulated Mn in all tissues and the accumulation increased with increasing Mn concentration in the culture solution. When exposed to hydroponic solution with 5 μM Mn (corresponding to 0.28 mg Mn/kg), the Mn content in *Eucalyptus grandis* × *E*. *urophylla* tissues were: leaf (595 mg/kg) > stem (157 mg/kg) > root (52 mg/kg). When treated with 500 μM Mn (corresponding to 28 mg/kg), the stems (3662 mg/kg) accumulated a similar amount of Mn as leaves (3792 mg/Kg), and both were approximately 15 times higher than roots (249 mg/Kg). When exposed to elevated Mn levels, especially 10,000 and 20,000 μM (corresponding to 549.4 and 1098.8 mg/Kg), we unexpectedly observed that the stems accumulated much higher Mn (29,018 and 52,540 mg/Kg) than the leaves (5,676 and 11,715 mg/Kg) and roots (1,965 and 8,459 mg/Kg). These observations indicate that young *Eucalyptus grandis* × *E*. *urophylla* plants accumulate more Mn in the stems when exposed to extremely toxic Mn levels to protect the young leaves, which is an important site for photosynthesis. Nevertheless, when the plants mature and have enough leaves to increase their Mn storage capacity and Mn detoxification, the Mn is transported and stored in the leaves, which explain why the leaf Mn concentration (13549 mg/kg) was much higher than that in the stems (5231 mg/kg) of the 1–2 years old *Eucalyptus grandis* × *E*. *urophylla* collected at the mining area. This could imply that the Mn stem-accumulation of *Eucalyptus grandis* × *E*. *urophylla* during its early development is a useful strategy to protect seedlings from Mn stress. However, while our results are suggestive, it is unclear if the development stage has a strong effect on metal accumulation and additional experiments on different plant species are warranted.

### Mn distribution in the anatomical structures of leaves and stems

**Fig 4:**
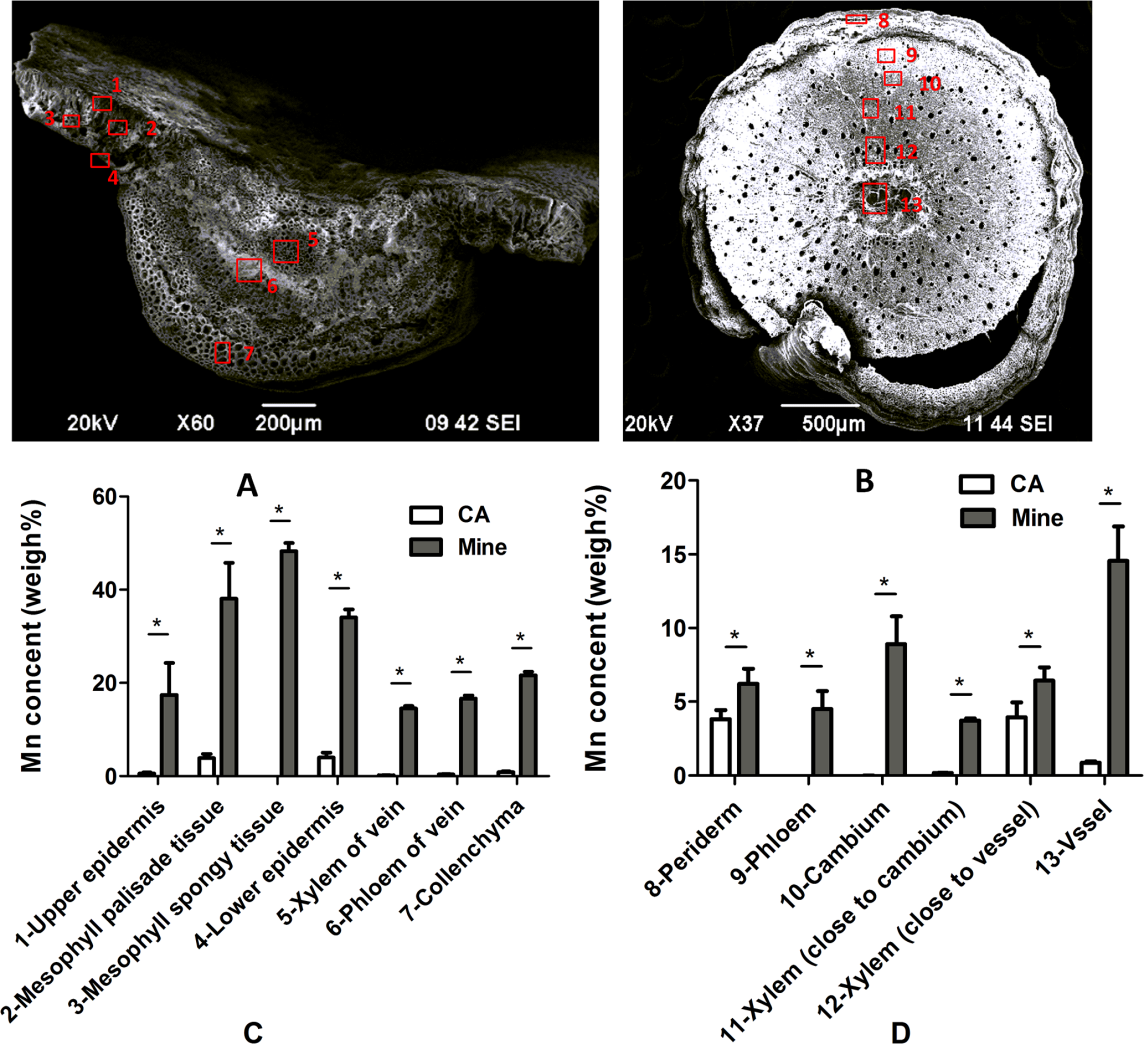
Anatomical structure of the *Eucalyptus grandis* × *E*. *urophylla*: a leaf from the mining area (A) including the upper epidermis (1), palisade (2), sponge tissue (3), lower epidermis (4), xylem of vein (5), phloem of vein (6) and collenchyma (7); stem from mining area (B) including the periderm (8), phloem (9), cambium (10), xylem near the cambium (11), xylem near the vessel (12), vessel (13). (C) Distribution of Mn in the leaf cross-section. (D) Distribution of Mn in the stem cross-section. The empty bar represented samples from the control area (CA) and the filled gray bar represented samples from the mine tailing area (Mine). Data represents mean ± SE. (n = 3). (The asterisks on the bars indicate significant differences after t-test statistical analyses, *p < 0.05).

Using SEM microanalysis, we observed the anatomical structures of the leaves and stems of *Eucalyptus grandis* × *E*. *urophylla* using cross sections of samples collected from the control area and the Mn site. SEM pictures of the Mn site samples are shown in (Fig 4A and 4B). The red square points were selected for X-ray analysis of elemental content. The weight percent of chosen elements, including Na, Mg, Si, P, S, Cl, K, Ca and Mn in mature leaf and stem tissues of *Eucalyptus grandis* × *E*. *urophylla* from the control area are shown in (Fig 4C and 4D). These results suggest that Mn accumulated in the mesophyll palisade (3.86%) and lower epidermis (4.03%), but less accumulation was observed in other leaf tissues, such as the upper epidermis (0.57%), mesophyll spongy (0.02%), xylem of vein (0.11%), phloem of vein (0.45%). While Mn was dramatically higher in all leaf tissues and was distributed across the entire leaf in samples collected from the Mn site (Spongy tissue 48% > Palisade tissue 38% > Lower epidermis 34% > Upper epidermis 17%), Mn accumulation in the main vein vasculature was lower than in the mesophyll and no dominant sinks were identified (Collenchyma 22% > Phloem of vein 16.8% > Xylem of vein 15.1%). Unlike the leaf, the main Mn sink in the stems was the vascular tissue in samples collected from the Mn site. In stems from the control area, Mn accumulated in the xylem close to vessel (3.95%) and periderm (3.82%); the phloem (0.05%), cambium (0.01%), the xylem close to cambium (0.17%) and vessel (0.86%) had low Mn accumulation based on weight percent. In stems from the Mn site, the highest Mn was found in the vessel (14.6%), followed by the cambium (8.91%), the xylem close to the vessel (6.43%), periderm (6.21%), phloem (4.5%), and the xylem close to the cambium (3.73%).

Compartmentalization of foliar Mn is quite different among metal hyper-accumulators. Large palisade mesophyll cells having high vacuolar Mn content are reported to be the main site of Mn storage for some hyper-accumulators, such as *Phytolacca Americana* L. and *Acanthopanax sciadophylloides* L. [45–49]. One hypothesis is that the nutritional importance of Mn might drive its deposition in the mesophyll. However, in species like *Maytenus fournieri* L., high deposition has been observed in non-photosynthetic cells of the upper-epidermis, which can be highly vacuolated or differentiate into double-layers [46, 50]. Moreover, Mn was found to be almost evenly sequestered across the entire leaf cross section of *Gossia*. *Amplexicaulis* L, in the floating lamina of *Trapa natans* L, and throughout the photosynthetic tissues as well as in the palisade and spongy mesophyll of *Gossia hillii* L [46, 47]. Furthermore, Mn was localized in the trichomes of *Alyssum murale* L. and the trichome base of sunflower (*Helianthus annus* L.) [51, 52]. In our study, Mn was distributed in the entire leaf cross-section of *Eucalyptus grandis* × *E*. *urophylla*, and was relatively high in the photosynthetic palisade and spongy tissue, both of which are highly vacuolated and may play an important role in storing excess Mn [46]. Mn is an important plant nutrient for photosynthesis, and therefore can be regarded as ‘less toxic’ than other trace heavy metals such as Cd and As, this may be the reason Mn can be stored in many different parts of the leaf. Still, it is difficult to explain why different hyperaccumulators have different Mn distribution patterns. Answering this question requires a clearer understanding of the function and distribution of Mn transporters in the Mn hyperaccumulators. In Arabiposis and rice, people have identified several Mn influx and efflux transporters, including members of the NRAMP (natural resistance associated macrophage protein), YSL (yellow stripe-like), ZIP (zinc regulated transporter/iron-regulated transporter [ZRT/IRT1]-related protein), CAX (cation exchanger), CCX (calcium cationex changers), CDF/MTP (cation diffusion facilitator/metal tolerance protein), P-type ATPases and VIT (vacuolar iron transporter) protein families. These transporters localized in cell membranes, such as plasma membrane, vacuole and chloroplast, and are responsible for Mn uptake and distribution in cells [1]. However, only YSL4 and YSL6 has been reported to export Fe^2+^ from vacuoles and chloroplasts in *Arabidopsis thaliana* L. and the *ysl4ysl6* double knock-out mutant is more tolerant to excess Mn than wild type plants, but the physiological role of AtYSL4 and AtYSL6 in Mn homeostasis remains unclear [1, 53]. Additionally, these transporters are differently expressed in various tissues. Thus, the expression level and tissue-pattern of these transporters will undoubtedly cause dramatic differences in Mn distribution at the cellular and tissue levels of different plants. Unfortunately, to our knowledge, there have been no studies focusing specifically on the Mn transporters in Mn hyperaccmulators.

A decrease in Mn concentration from the midrib to the margin was observed within leaves of *P*. *acinosa* L., suggesting that Mn is transported along with the transpiration stream [54]. However, little data is available regarding Mn distribution in stems. This study provides in-vivo Mn-distributions showing a relatively high Mn accumulation in the stem xylem vessel, which is an important pathway of the transpiration stream, and suggests that Mn is mainly transported through the xylem vessel to the leaf and distributed to other parts of the stem. Depositing heavy metals can thicken the vascular wall, and the deposition of Mn and other elements onto the apoplast (leaf surface and vascular wall) could significantly reduce the toxic effects of excess Mn in the symplast [39]. In *Eucalyptus grandis* × *E*.*urophylla*, the major Mn sink in the stem was primarily the stem vessel, which is composed of dead cells and the cell wall composed primarily of polysaccharides and proteins. They have numerous functional groups, such as –COOH, –OH and –SH as well, playing a crucial role in metal binding [55]. In this regard, the stem is important for Mn detoxification not only as a transportation system but also a physical barrier against toxic Mn, especially in the early development stages of *Eucalyptus grandis* × *E*. *urophylla*.

### Subcellular distribution of Mn

**Fig 5:**
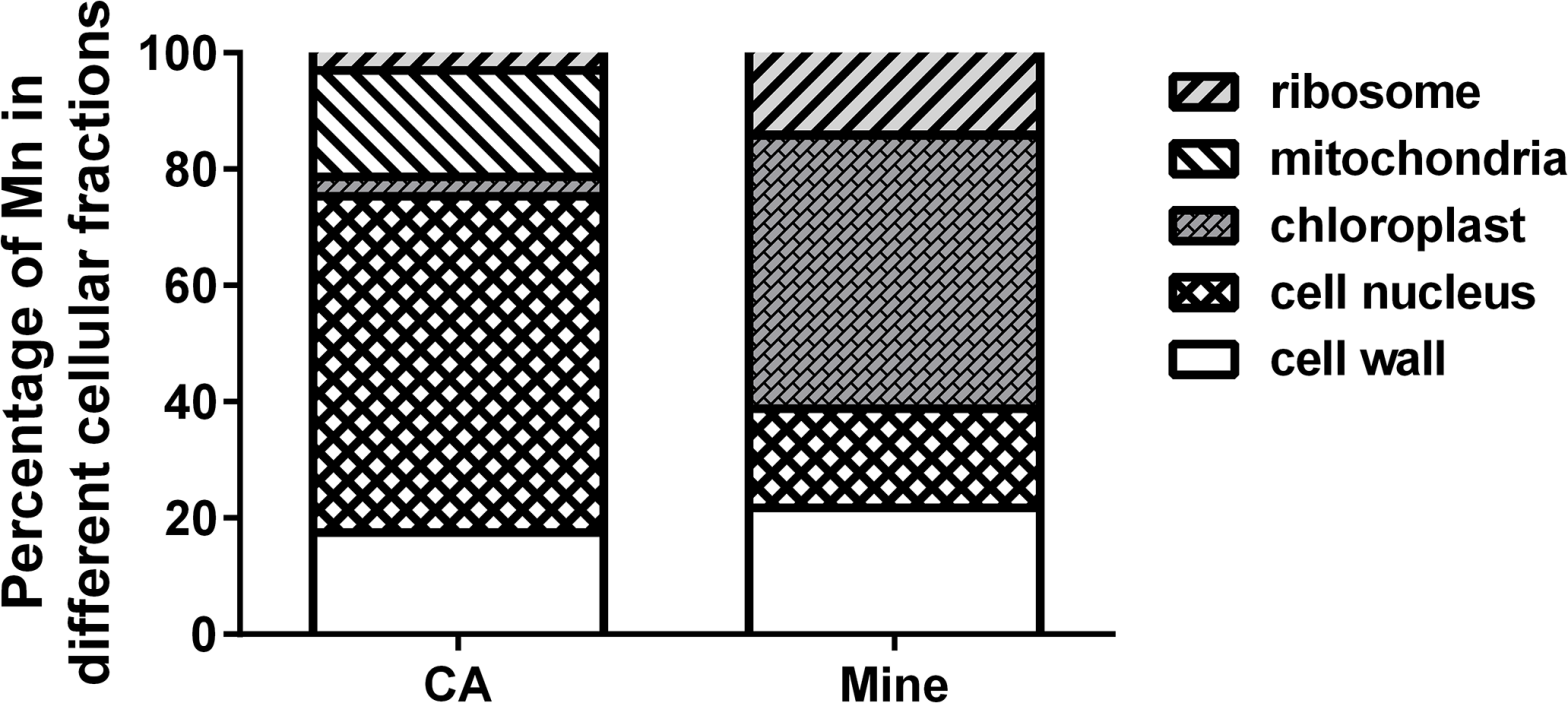
Subcellular distribution of Mn. CA represents samples from the control area, and Mine represents samples from the mine tailing area. Data represents mean ± SE. (n = 3).

We also investigated the subcellular distribution of Mn in the leaves of *Eucalyptus grandis* × *E*. *urophylla* (Fig 5). Compared to whole leaf Mn, the cell wall Mn fraction in samples collected from the Mn site accounted for 21.81% which was slightly higher than in samples from the control area (18.17%). Furthermore, the ribosome accounted for about 14.06%, which is about 4 times higher than in the control, and a large proportion of Mn was stored in the chloroplast (46.98%), which comprised almost half of the total Mn in the leaf from the Mn site, while the Mn proportion in the mitochondria decreased to 0.17%. (Fig 5). The vacuole, chloroplast and cell wall have all been reported to be the Mn pools in various plant cells [39, 54, 56]. The majority of Mn was found in the chloroplast of *Eucalyptus grandis* × *E*. *urophylla* sampled from the Mn site, which supports a recently reported novel detoxifying strategy and indicates that excess Mn in the chloroplast was detoxified by being deposited in the starch granule [39]. It is possible that the starch granules also help *Eucalyptus grandis* × *E*. *urophylla* detoxify excess Mn in the chloroplast.

### Mn in the water-soluble fractions from leaves and stems

**Fig 6:**
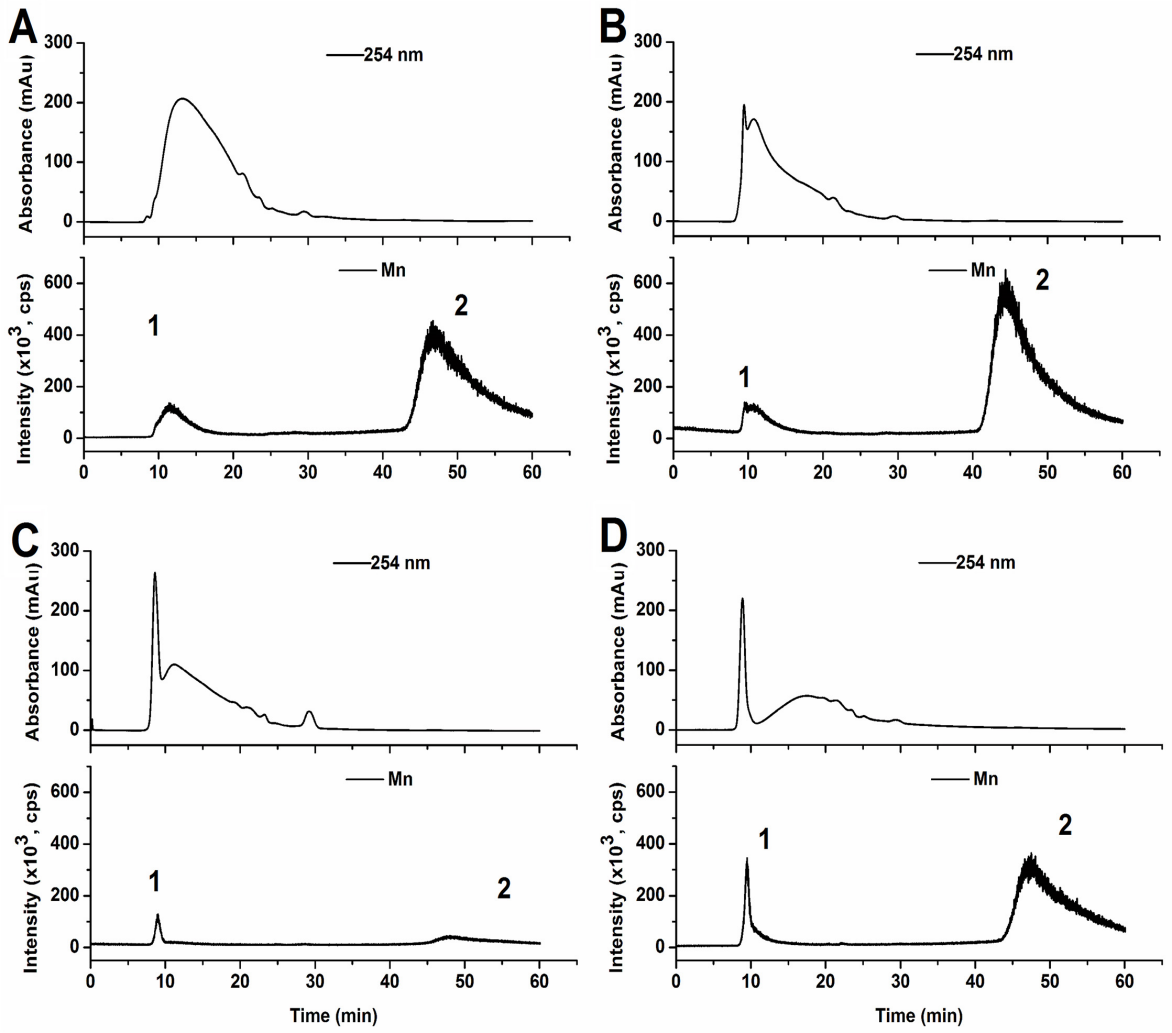
SEC/UV/ICPMS chromatograms of the water-soluble fractions from leaves and stems of *Eucalyptus grandis* × *E*. *urophylla*. The leaves (A) and stems (C) from the control area; the leaves (B) and stems (D) from the mining area; Peak 1, Mn associated with high molecular weight proteins; peak 2, Mn associated with low molecular weight compounds.

The water-soluble fractions collected from leaves and stems were used for size-exclusion chromatography (SEC) coupled with UV detection (λ = 254 nm) and ICP-MS in sequence. SEC/UV/ICP-MS chromatograms of the water-soluble fractions from leaves of the control and Mn site are shown in (Fig 6A and 6B). Two major peaks containing Mn were observed based on the Mn ICP-MS signals. From the retention time of the different molecular weight standards under the same chromatographic conditions, peak 1 at around 11 min is most likely to be proteins having molecular weights larger than the ovalbumin (OVA, 45000 Da, elution time was 13.8 min); and the compounds in peak 2 at 47 min have molecular weights less than our GSH standard (307 Da, elution time was 28.3 min). These results suggest that Mn in the water-soluble fraction of leaves is associated with both high molecular weight (HMW) proteins and low molecular weight (LMW) compounds. The Mn ICP-MS intensities of either peak 1 or 2 (Fig 6A and 6B) from the water-soluble fraction of the leaves from the Mn site are higher than those from the control site, and peak 2 is higher than peak 1, implying that Mn is significantly associated with LMW compounds. It should be noted that the amount of Mn associated with HMW proteins (peak 1) in samples from the Mn site and the control samples were quite similar (around 1.5 × 10^5^ cps), while Mn associated with LMW compounds (peak 2) in samples from the Mn site remarkably increased, suggesting that excess Mn from the Mn site tends to bind the low molecular weight compounds rather than the HMW proteins in the leaves. In the case of the stems (Fig 6C and 6D), although similar phenomena were observed in that the amount of Mn associated with the HMW proteins (peak 1) and the LMW compounds (peak 2) in the water-soluble fraction of the leaves from the Mn site were higher than those of the control, the amount of Mn associated the HMW proteins and LMW compounds from the control were at same level when considering the peak area, the peak 1 and 2 in (Fig 6C). In comparison, Mn associated with the LMW compounds from stems collected at the Mn site significantly increased, the peak 2 in (Fig 6D) but that with the HMW proteins did not. These results further support the idea that excess Mn in the stems, like in the leaves from the Mn site, prefer to bind LMW compounds rather than proteins, which, in turn, means that the LMW compounds play a more important role in mitigating Mn stress.

Vacuolar compartmentalization or complexion of heavy metals by organic ligands has been reported as the main detoxification mechanisms in plants [57], for example, in *Phytolacca americana* L, a hyper-accumulator that can accumulate large amounts of cadmium and Mn. Cd treatments increased phytochelatin synthesis, however excess Mn treatments did not result in a significant increase in non-protein thiols or phytochelatin concentrations in the leaves or roots, but did increase the oxalic acid concentration in the leaves [58]. Our results indicate that LMW compounds in the water-soluble fractions of the leaves and stems of *Eucalyptus grandis* × *E*. *urophylla* played a significant role in detoxifying excess Mn in addition to the HMW proteins; however, the exact identity of the LMW compounds and the HMW proteins requires further investigation.

### Interaction of Mn with subcellular proteins in the leaves

**Fig 7:**
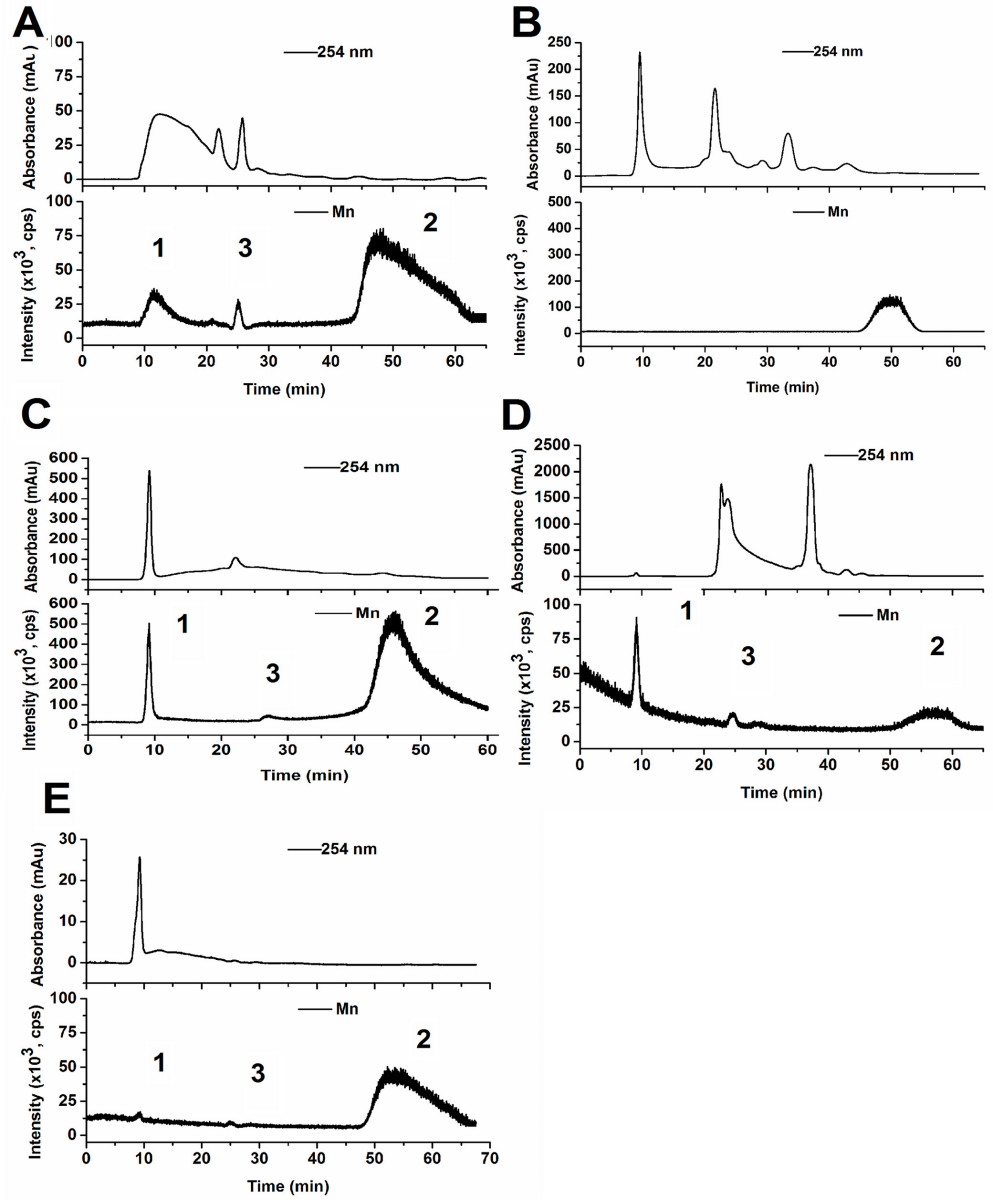
Elution profiles of leaf subcellular soluble proteins with Size-exclusion columns coupled to ICP-MS. (Leaf samples all from the mining area.) Covalently-bound cell wall protein (A) and ionically-bound cell wall protein (B); Chloroplast PSI(C); Chloroplast PSII (D) and Vacuole protein (E). 1, high molecular weight chelate; 2, middle molecular weight chelate; 3, low molecular weight.

Plant cell walls provide not only physical barriers against toxic heavy metals but also are the primary site for signal perception of environmental stresses [59]. The main components of cell walls are polysaccharides (90%) and proteins (less than 10%). The cell wall proteome is reported to be regulated by Cu, and apoplast proteins play major roles in many physiological events, such as cell wall polysaccharide remodeling, cell metabolism process and antioxidant defense pathway. Apoplast proteins related to polysaccharide remodeling and the apoplastic peroxidases were induced by Mn stress, too [59, 60]. However, evidence of Mn-binding cell wall proteins is scarce. Cell wall proteins can be divided into two major categories based on their chemical and physical association with cell walls, namely covalently bound and ionically bound proteins in the glycan network. In the cell wall protein fractions, we found HMW proteins (peak 1, *t* = 25 min, MW > 45K Da), LMW organic compounds (peak 2, *t* = 47 min, MW much smaller than our standard GSH, which is 307 Da) and a new Mn-containing peak (peak 3, retention time *t* = 25 min, between peak 1 and peak 2) (Fig 7A) This small peak is thought to contain proteins of middle molecular weight. This covalently bound protein could not be identified in the water-soluble fraction (Fig 6) because cellulase and pectinase were not applied. Interestingly, we found only Mn bound to the LMW organic compounds (peak 2) in ionically-linked cell wall protein fractions (Fig 7B). It was reported that Mn can induced three clusters of apoplast proteins, 25–30 KDa proteins, including a pathogenesis-related protein, 32 KDa apoplastic peroxidases and 40–45 KDa proteins, including chitinases and glucanases. Whether these proteins can bind to Mn is currently unknown [61]. It is possible that these Mn-associated proteins play a role in Mn detoxification; however, we could not identify their exact molecular weight and structure in this study. Future experiments using high resolution mass spectrometry will be required to further address this question.

Manganese is critical for the photosynthetic apparatus [62], including the PS II core and the Mn_4_CaO_5_ cluster that catalyzes the splitting of water and the production of O_2_ [2, 63, 64]. This could partially explain why the majority of the Mn accumulated in the chloroplast of *Eucalyptus grandis* × *E*. *urophylla*. PS II complex assembly is a complex process and happens in several membranes of the chloroplast. It was reported [65] that Mn^2+^ is taken up into the periplasmic space and incorporated by the PS II assembly factors Prat A complex (36 KD), and then Mn-Prat A would is delivered to the chloroplast plasma membrane, binding to the precursor form of D1 (pD1, 45 kDa) and forming a Mn-Prat A-pD1 complex. Next, YCF48 is joined and converted into the RC47 complex (RC47-Mn) and several low-molecular-mass PS II subunits bind CP47, resulting in a functional PS II-Mn-core. Among these process, proteins have a molecular weight varying from 12–76 KD were added step by step [2]; however during the protein extraction and elution steps, some loosely bound sub-units of PS II are broken apart from the unit. The results obtained in this study also indicate that Mn was abundant and accounted for 46.89% of the total Mn in the leaf chloroplasts of *Eucalyptus grandis* × *E*. *urophylla* taken from the Mn site. In the isolated PS II fraction, the Mn-containing HMW proteins (peak 1) and the LMW organic compounds (peak 2) were found as well as medium molecular weight proteins (peak 3) that had been observed in the cell wall protein fraction (Fig 7A). Similar observed were made in the chloroplast PS I fraction, although the amount of Mn associated with the LMW organic compounds was very high. These results might imply that Mn interacted with similar proteins and small organic molecules in the cell wall and chloroplast of the leaves of *Eucalyptus grandis* × *E*. *urophylla* collected in the Mn site of the mining area. It should be noted that the exact identity of these proteins requires further investigation. For the isolated vacuoles, the Mn-containing LMW organic compounds (peak 2) were found to be the predominant component, while the Mn-containing HMW proteins (peak 1) and the medium molecular weight proteins (peak 3) were low abundance. These results suggest that most of the proteins associated with Mn are fixed in the organelle structure and cannot be translocated into the vacuole, on the other hand, the LMW organic compounds associated with Mn could easily be transported into the vacuole and play an very important role in Mn detoxification.

### Organic acids in the leaves and stems

**Fig 8:**
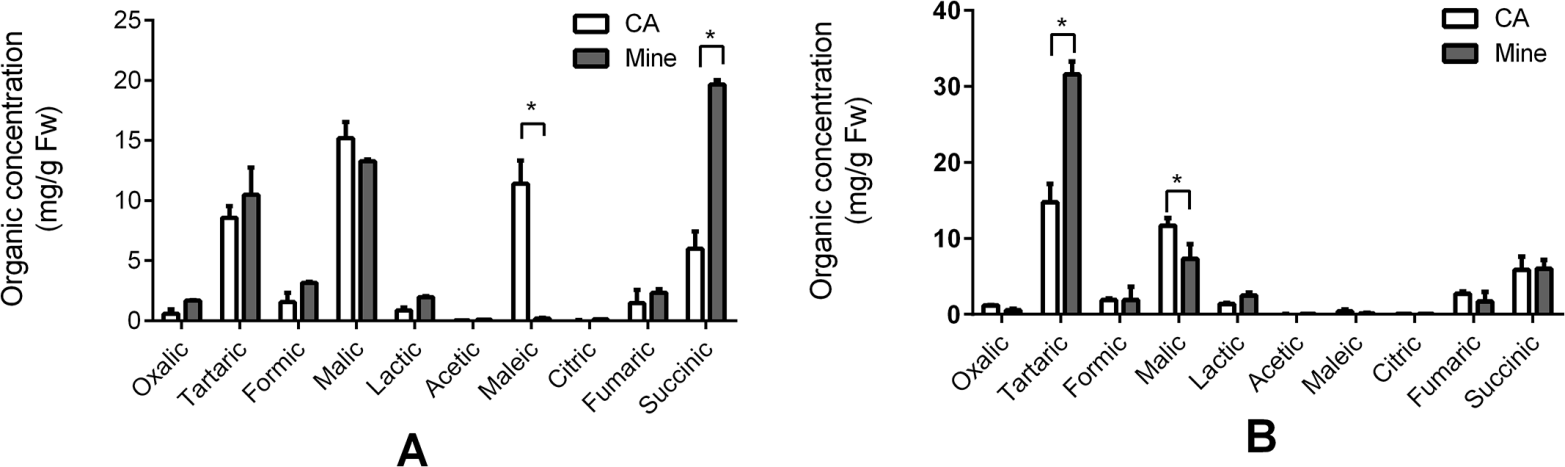
Concentrations of organic acids in leaves (A) and stems (B). The empty bar represents samples from the control area (CA) and the filled gray bar represents samples from the Mn site of the mine tailing area. Data represents mean ± SE. (n = 3). The asterisks on the bars indicate significant differences after t-test statistical analyses, *p < 0.05.

We extracted organic acids from the leaf and stem samples from both the control and Mn site with liquid nitrogen and dilute HCl. The acid-extractable fractions, which were believed to contain organic acids, were subjected to RP-HPLC. The results obtained (Fig 8A) indicate that leaf samples from the control area contain higher amounts of malic acid (15.19 ± 1.36 mg/g FW), maleic acid (11.4 ± 1.91), tartaric acid (8.57 ± 0.96), and succinic acid (5.98 ± 1.46), but lower fumaric (1.46 ± 1.12), formic (1.56 ± 0.77 mg/g FW) and oxalic acids (0.60 ± 0.37 mg/g FW). Compared with leaf samples from the control site, a greater than three fold increase in succinic acid (19.65 ± 0.38 mg/g FW) was found in leaf samples from the Mn site along with a slight increase in other organic acids. Surprisingly, we observed a decrease in maleic acid from (11.4 ± 1.91 mg/g FW) in the control to undetectable levels in samples collected from the Mn site along with a small decrease in malic acid. It should be noted that both acetic acid and citric acid were found at very low levels in not only the control leaves but also leaves from the Mn site. Unlike in the leaf samples, tartaric acid was the most abundant organic acid found in the stem sample extracts (Fig 8B). It increased significantly by about 2 fold in the Mn site stem samples (31.55 ± 1.69 mg/g FW) compared to the control area samples (14.76 ± 2.39 mg/g FW). However, malic and succinic acid concentrations decreased to (7.32 ± 1.92) and (6.03 ± 1.11 mg/g FW) in the leaf samples. Oxalic (0.57 ± 0.16 mg/g FW), formic (1.88 ± 1.73 mg/g FW), lactic (2.48 ± 0.36 mg/g FW), acetic (0.04 ± 0.001 mg/g FW), maleic (0.13 ± 0.10 mg/g FW) and citric acid (0.08 ± 0.001 mg/g FW) in stems from the Mn site were all lower than in samples from the control site. These results indicated that Mn exposure caused a dramatic decrease in maleic acid and an increase in succinic acid in the leaves but had smaller affects on other organic acids; however, tartaric acid did increase significantly in the stems.

**Fig 9:**
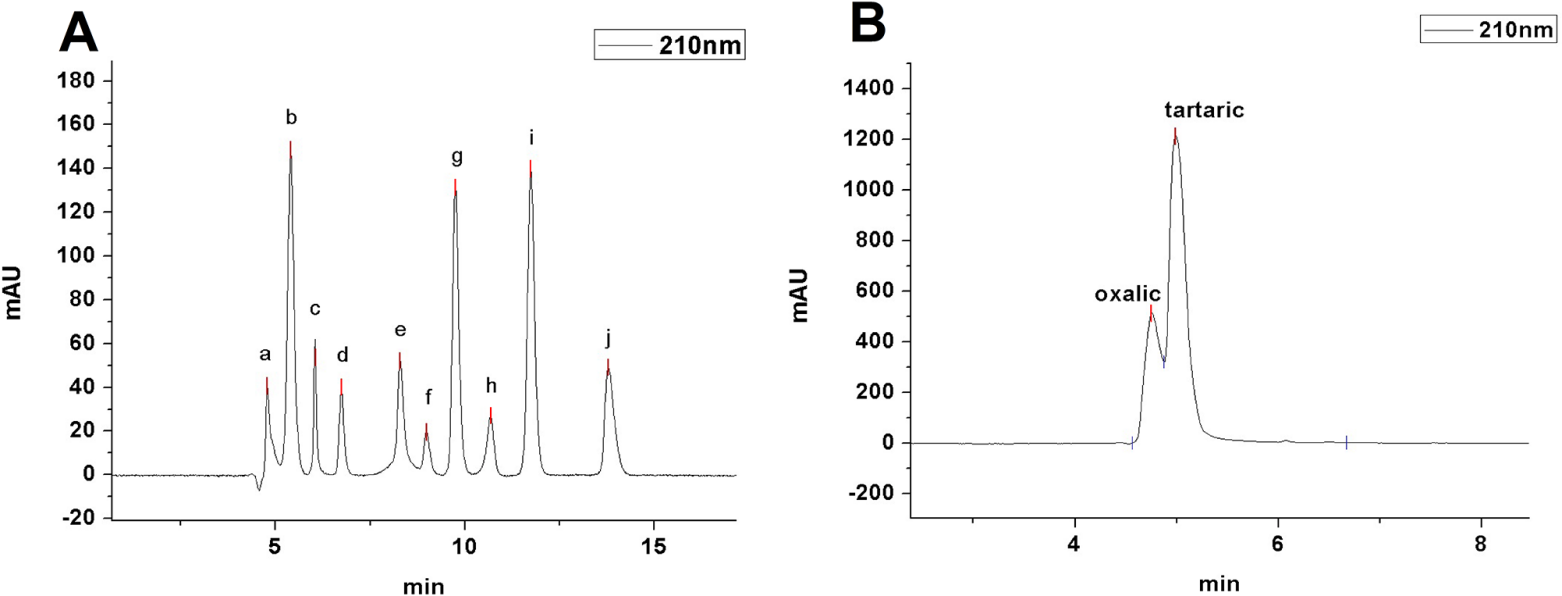
Typical chromatogram of ten organic acid standards by RP-HPLC (A). a, oxalic acid; b, tartaric acid; c, formic acid; d, malic acid; e, lactic acid; f, acetic acid; g, maleic acid; h, citric acid; i, fumaric acid; j, succinic acid. (B) RP-HPLC of the small molecular-Mn complexes in the leaf sample peak 2 (Fig 6B).

When compared with the elution time of the organic acid standards (Fig 9A), the low-molecular weight Mn fraction peak 2 in (Fig 6B) has a similar elution time as oxalic and tartaric acid in the leaves (Fig 9B). Unexpectedly, Mn did not bind to succinic acid, which was the highest abundance acid in the leaves. Although succinic acid metabolism was affected by Mn stress, the role of succinic acid remains unclear. Mn stress greatly increased the tartaric acid concentration in the stems, which explains why the Mn stem-accumulation of *Eucalyptus grandis* × *E*. *urophylla* during early development is a helpful strategy to protect the seedlings from Mn stress.

## Conclusions

A inducible Mn-hyperaccumulator *Eucalyptus grandis* × *E*. *urophylla* was identified during a field survey in southern China in July 2010. This plant can accumulate as much as 13,549 mg/kg DW Mn in its leaves. SEM X-ray microanalysis suggested that Mn is distributed throughout the entire leaf and stem cross-section, especially in photosynthetic palisade, spongy mesophyll tissue, and stem xylem vessel; SEC/ICP-MS and RP-HPLC studies led us to speculate that Mn might be associated with relatively high molecular weight proteins and low molecular weight organic acids, such as tartaric acid to prevent Mn toxicity. We have provided experimental evidence that proteins and organic acids play important roles in Mn detoxification in *Eucalyptus grandis* × *E*. *urophylla*. The Mn-speciation profile obtained for the first time in different cellular organelles of *Eucalyptus grandis* × *E*. *urophylla* suggested that different organelles have their own accumulating abilities and unique mechanism for Mn-detoxification. Tartaric acid in the stem induced by Mn stress allows the stem to store more Mn during early developmental stages, and facilitates Mn translocation by transpiration through xylem to the leaves to further distribute Mn throughout the leaf tissues. The information gained in the current study and similar mechanistic studies are important for future applications of *Eucalyptus grandis* × *E*. *urophylla* as a phytoremediation tool for Mn polluted soils.
